# High Expression of Testes-Specific Protease 50 Is Associated with Poor Prognosis in Colorectal Carcinoma

**DOI:** 10.1371/journal.pone.0022203

**Published:** 2011-07-12

**Authors:** Lei Zheng, Ganfeng Xie, Guangjie Duan, Xiaochu Yan, Qianwei Li

**Affiliations:** 1 Department of Nuclear Medicine, Southwest Hospital, Third Military Medical University, Chongqing, China; 2 Department of Oncology and Southwest Cancer Center, Southwest Hospital, Third Military Medical University, Chongqing, China; 3 Department of Pathology and Southwest Cancer Center, Southwest Hospital, Third Military Medical University, Chongqing, China; University of Chicago, United States of America

## Abstract

**Background:**

Testes-specific protease 50 (TSP50) is normally expressed in testes and abnormally expressed in breast cancer, but whether TSP50 is expressed in colorectal carcinoma (CRC) and its clinical significance is unclear. We aimed to detect TSP50 expression in CRC, correlate it with clinicopathological factors, and assess its potential diagnostic and prognostic value.

**Methodology/Principal Findings:**

TSP50 mRNAs and proteins were detected in 7 CRC cell lines and 8 CRC specimens via RT-PCR and Western blot analysis. Immunohistochemical analysis of TSP50, p53 and carcinoembryonic antigen (CEA) with tissue microarrays composed of 95 CRCs, 20 colorectal adenomas and 20 normal colorectal tissues were carried out and correlated with clinicopathological characteristics and disease-specific survival for CRC patients. There was no significant correlation between the expression levels of TSP50 and p53 (*P* = 0.751) or CEA (*P* = 0.663). Abundant expression of TSP50 protein was found in CRCs (68.4%) while it was poorly expressed in colorectal adenomas and normal tissues (*P*<0.0001). Thus, CRCs can be distinguished from them with high specificity (92.5%) and positive predictive value (PPV, 95.6%). The survival of CRC patients with high TSP50 expression was significantly shorter than that of the patients with low TSP50 expression (*P* = 0.010), specifically in patients who had early-stage tumors (stage I and II; *P* = 0.004). Multivariate Cox regression analysis indicated that high TSP50 expression was a statistically significant independent risk factor (hazard ratio  = 2.205, 95% CI = 1.214–4.004, *P* = 0.009).

**Conclusion:**

Our data demonstrate that TSP50 is a potential effective indicator of poor survival for CRC patients, especially for those with early-stage tumors.

## Introduction

The testes-specific protease 50 (TSP50) gene was discovered from a human testes cDNA library on a hypomethylated DNA fragment isolated from human breast cancer cells via the methylation sensitive-representational difference analysis technique [1]. It encodes a threonine protease which is homologous to serine proteases, but its crucial catalytic triad has a substitution of threonine at the serine residue site [2]. *TSP50* is normally and specifically expressed in the spermatocytes of testes, abnormally activated and expressed in most (more than 90%) breast cancer biopsies, and negatively regulated by the *p53* gene, which can in turn promote tumorigenesis [2]–[4]. Further, previous investigations found that basic fibroblast growth factor (bFGF) could downregulate *TSP50* expression via the ERK/Sp1 pathway due to *TSP50* gene promoter containing Sp1 binding site [5], [6]. Most importantly, recent studies reported that knockdown of *TSP50* gene expression could inhibit cell proliferation, colony formation and migration, induce cell apoptosis, and enhance cell sensitivity to doxorubicin [7], and the underlying molecular mechanisms might be related to activation of the NF-κB signaling pathway [8]. These results imply that the *TSP50* gene should be an oncogene, and the TSP50 protein might be a biomarker for human breast cancer. Based on the information above, TSP50 is considered as a cancer/testis antigen (CTA) [3], [9]. Many CTAs, such as MAGEA1, NY-ESO-1, SYCP1, BRDT, HOM-TES-85, NFX2 and SSX-1, are expressed in various human cancers [10]–[17]. However, to our knowledge, there is no report that TSP50 has been detected in other human malignancies except breast cancer.

Previous studies have demonstrated that the *TSP50* gene promoter's DNA methylation status most likely control the gene expression in different types of tissues [18]. DNA methylation is associated with *TSP50* gene silencing in many normal tissues such as breast, lung and kidney. Conversely, DNA demethylation is associated with elevated levels of *TSP50* gene expression in the testes and breast cancer [1], [18]. Moreover, global hypomethylation is common and prominent in colorectal carcinoma (CRC) as compared to normal colorectal tissue [19]–[21], and some other CTAs have already been detected in CRC [22]–[24]. Therefore, we speculated that TSP50 could be expressed in CRC.

To date, the expression state of *TSP50* gene in CRC and its relationship with clinicopathological/prognostic significance is unknown. We aimed to analyze the expression status of TSP50 in CRCs compared with colorectal adenomas and normal tissues, determine its relationship with clinicopathological parameters, and investigate its prognostic value for CRC patients based on tumor stage (early and advanced stage). In addition, p53 protein expression was examined to investigate its correlation with TSP50 expression in CRCs, and the prognostic significance of carcinoembryonic antigen (CEA), a well established prognostic factor for CRC, was analyzed to verify the reliability of this cohort of CRC patients. We found that TSP50 could be a very useful predictor for unfavorable prognosis in patients with CRC.

## Results

### Detection of TSP50 expression in the CRC cell lines and tissues

**Figure 1:**
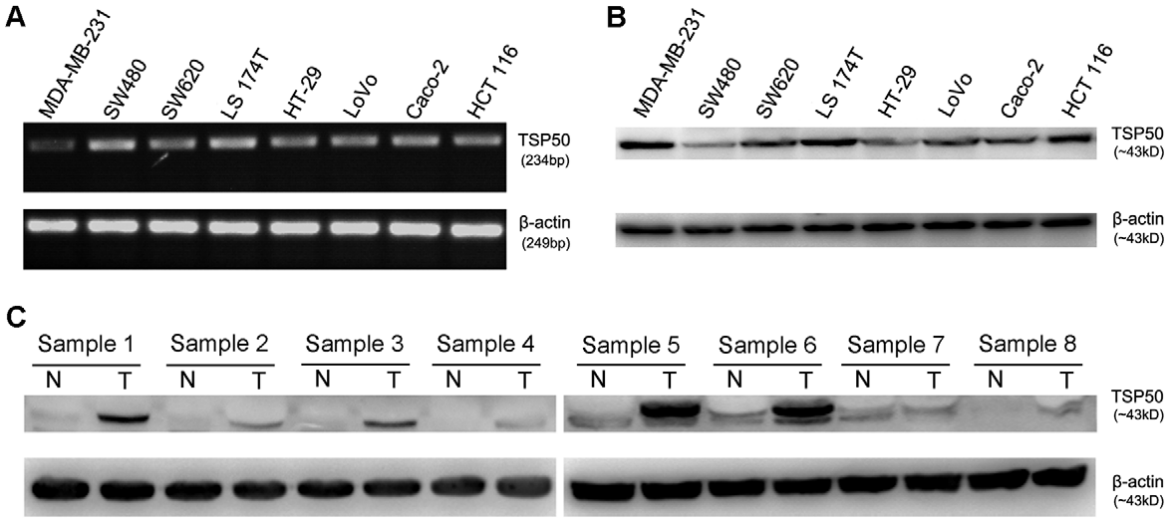
Expression of TSP50 in CRC cell lines and tissue specimens. (A) RT-PCR of TSP50 expression in the 7 CRC cell lines; (B) Western blot analysis of TSP50 expression in the 7 CRC cell lines; (C) Western blot analysis of TSP50 expression in 8 CRC specimens (T) and adjacent normal colorectal specimens (N) paired from the same patient. Total RNA and protein from the breast cancer cell line MDA-MB-231 served as the positive controls. β-actin served as internal control.

Aberrant expression of TSP50 was detected in all the 7 CRC cell lines by RT-PCR and Western blot analysis (Figure 1A and B). Total RNA and protein from the breast carcinoma cell line MDA-MB-231 served as positive controls, and β-actin served as internal control. TSP50 was expressed in all the 8 CRC samples, and not or weakly expressed in the adjacent normal colorectal tissues (Figure 1C). TSP50 expression levels were obviously higher in most CRC samples than those in the adjacent normal colorectal tissues.

### Immunohistochemical analysis of TSP50 expression in colorectal normal tissues, adenomas and CRCs

**Figure 2:**
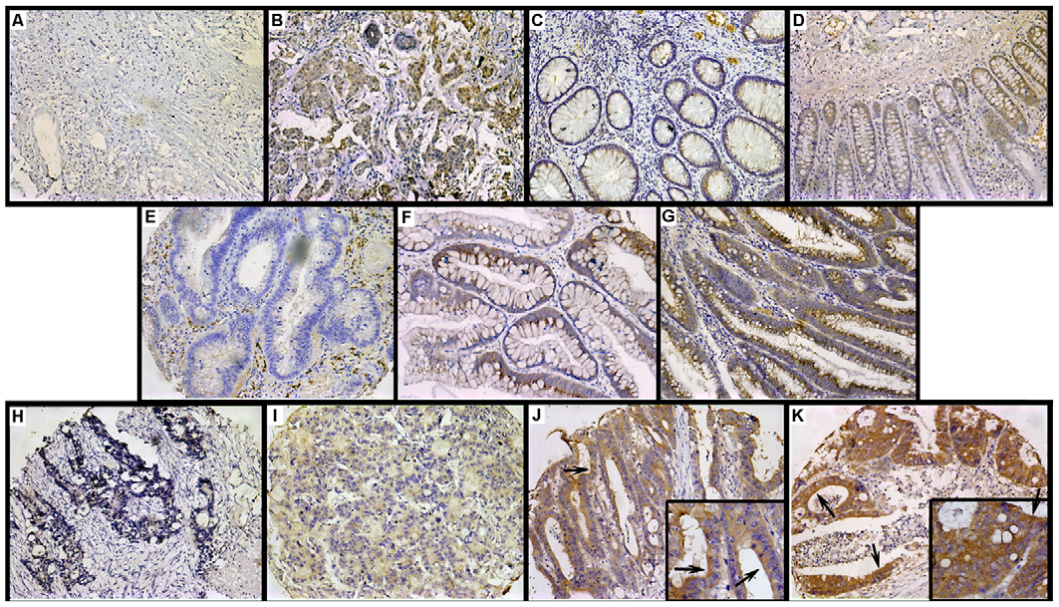
Immunohistochemical analysis of TSP50 in colorectal tissue microarrays. (A) The breast carcinoma section that was incubated with PBS served as negative control; (B) The breast carcinoma section that was incubated with antibodies to TSP50 served as positive control; (C–D) A sample of TSP50 expression levels in colorectal normal tissues: − in C and 1+ in D; (E–G) A sample of TSP50 expression levels in colorectal adenomas: − in E, 1+ in F and 2+ in G; (H–K) A sample of TSP50 expression levels in CRCs: − in H, 1+ in I, 2+ in J and 3+ in K; some CRC samples exhibited membrane staining (arrows in J and K). Original magnification, ×200 in A–K and ×400 in *inset*.

The breast carcinoma sections which were incubated with PBS or antibodies to TSP50 served as negative control (Figure 2A) or positive control (Figure 2B). TSP50 expression was variable: grade − and 1+ in the colorectal normal epithelium (Figure 2C and D); grade −, 1+ and 2+ in colorectal adenomas (Figure 2E–G); grade −, 1+, 2+ and 3+ in CRCs (Figure 2H–K). TSP50 proteins were observed predominantly in the cytoplasm, but exhibited in the membrane and cytoplasm of some CRC samples (Figure 2J and K, arrows). TSP50 expression levels in CRCs were significantly higher than those in colorectal normal tissues or adenomas (*P*<0.0001; Table 1).

**Table 1:** Relationship between TSP50 expression and type of colorectal tissues or expression status of p53 or CEA.

	TSP50 expression (%)				*P*
	−	+	++	+++	
**Tissue type**					
Normal	8 (40.0)	12 (60.0)	0 (0.0)	0 (0.0)	<0.0001^a^
Adenoma	4 (20.0)	13 (65.0)	3 (15.0)	0 (0.0)	
CRC	10 (10.5)	20 (21.1)	57 (60.0)	8 (8.4)	
**p53 expression**					
−	5 (5.2)	8 (8.4)	30 (31.6)	4 (4.2)	0.751^b^
+	2 (2.1)	2 (2.1)	10 (10.5)	1 (1.1)	
++	1 (1.1)	7 (7.4)	8 (8.4)	1 (1.1)	
+++	2 (2.1)	3 (3.2)	8 (8.4)	3 (3.2)	
**CEA expression**					
−	2 (2.1)	3 (3.2)	9 (9.5)	0 (0.0)	0.663^b^
+	2 (2.1)	8 (8.4)	15 (15.8)	1 (1.1)	
++	3 (3.2)	7 (7.4)	23 (24.2)	7 (7.4)	
+++	3 (3.2)	2 (2.1)	9 (9.5)	1 (1.1)	

a, Kruskal Wallis Test; b, Spearman's rho.

TSP50, testes-specific protease 50; CRC, colorectal carcinoma; CEA, carcinoembryonic antigen.

### Relationship between TSP50 expression and p53 or CEA expression

**Figure 3:**
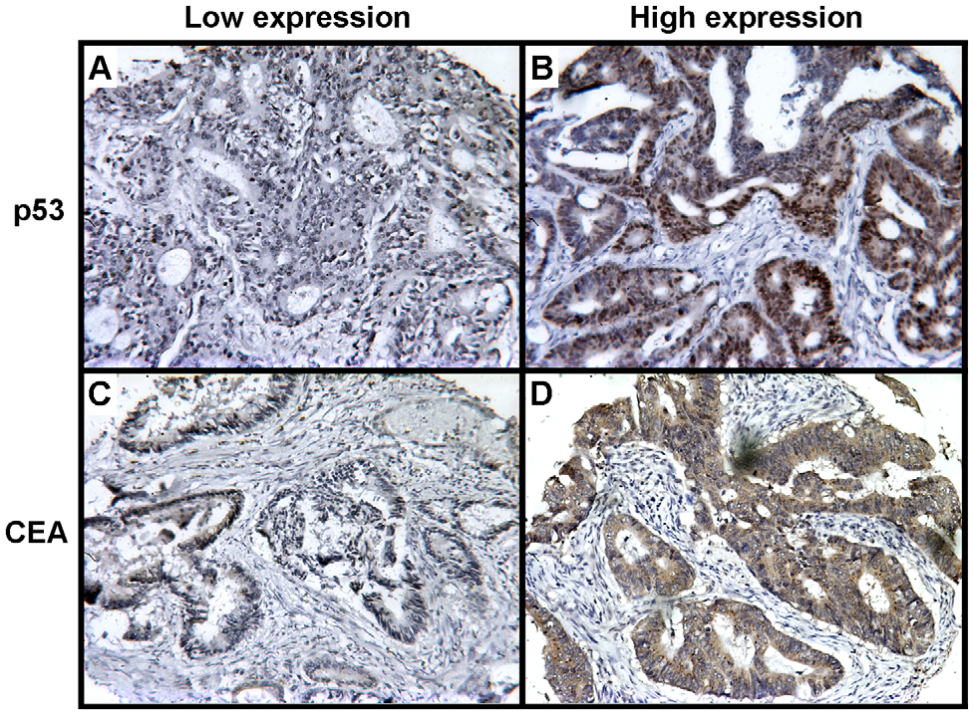
Representative immunohistochemical staining of p53 and CEA in CRCs. (A) Low expression of p53; (B) High expression of p53 in the nucleus of carcinoma cells; (C) Low expression of CEA; (D) High expression of CEA in the cytoplasm and membrane of carcinoma cells. Original magnification, ×200.

Expression of p53 protein was observed in the nucleus of carcinoma cells (Figure 3A and B), and the levels were variable: grade − in 47 (49.5%) cases, grade 1+ in 15 (15.8%) cases, grade 2+ in 17 (17.9%) cases, and grade 3+ in 16 (16.8%) cases of 95 CRCs (Table 1). CEA was expressed in the cytoplasm and/or membrane of carcinoma cells (Figure 3C and D), and its expression was variable: grade − in 14 (14.7%) cases, grade 1+ in 26 (27.4%) cases, grade 2+ in 40 (42.1%) cases, and grade 3+ in 15 (15.8%) cases (Table 1). There was no significant correlation between TSP50 and p53 or CEA expression (Table 1).

### Relationship between clinicopathologic features and TSP50, p53 or CEA expression

There was no significant association between TSP50 expression status in CRCs and all the clinicopathologic features including age, sex, depth of invasion, lymph node metastasis, and tumor location, size, stage and grade (Table 2). p53 overexpression was significantly associated with tumor location (*P* = 0.033), and CEA expression was negatively correlated with tumor grade (*P* = 0.020), but both were not related with other clinicopathologic characteristics analyzed (Table 2).

**Table 2:** Association between clinicopathologic features and survival or expression level of TSP50, p53 or CEA in CRCs.

Variables	Cases	Survival *P* (log-rank)	TSP50 expression (%)			p53 expression (%)			CEA expression (%)		
			Low	High	*P* (χ^2^)	Low	High	*P* (χ^2^)	Low	High	*P* (χ^2^)
Total number of patients	95		30 (31.6)	65 (68.4)		62 (65.3)	33 (34.7)		40 (42.1)	55 (57.9)	
**Age group, y**											
≤56	47	0.727	19 (40.4)	28 (59.6)	0.066	32 (68.1)	15 (31.9)	0.568	23 (48.9)	24 (51.1)	0.182
>56	48		11 (22.9)	37 (77.1)		30 (62.5)	18 (37.5)		17 (35.4)	31 (64.6)	
**Sex**											
Women	40	0.301	13 (32.5)	27 (67.5)	0.869	25 (62.5)	15 (37.5)	0.630	17 (42.5)	23 (57.5)	0.947
Men	55		17 (30.9)	38 (69.1)		37 (67.3)	18 (32.7)		23 (41.8)	32 (58.2)	
**Tumor location**											
Colon	40	0.450	9 (22.5)	31 (77.5)	0.104	31 (77.5)	9 (22.5)	**0.033**	16 (40.0)	24 (60.0)	0.723
Rectum	55		21 (38.2)	34 (61.8)		31 (56.4)	24 (43.6)		24 (43.6)	31 (56.4)	
**Tumor size, cm**											
≤4	50	0.602	16 (32.0)	34 (68.0)	0.926	29 (58.0)	21 (42.0)	0.117	21 (42.0)	29 (58.0)	0.983
>4	45		14 (31.1)	31 (68.9)		33 (73.3)	12 (26.7)		19 (42.2)	26 (57.8)	
**Depth of invasion**											
T_2_	20	0.080	4 (20.0)	16 (80.0)	0.174	15 (75.0)	5 (25.0)	0.566	9 (45.0)	11 (55.0)	0.305
T_3_	44		18 (40.9)	26 (59.1)		27(61.4)	17 (38.6)		15 (34.1)	29 (65.9)	
T_4_	31		8 (25.8)	23 (74.2)		20 (64.5)	11 (35.5)		16 (51.6)	15 (48.4)	
**Lymph node metastasis**											
N_0_	55	**<0.0001**	18 (32.7)	37 (67.3)	0.862*	35 (63.6)	20 (36.4)	0.569*	27 (49.1)	28 (50.9)	0.157
N_1_	26		7 (26.9)	19 (73.1)		19 (73.1)	7 (26.9)		10 (38.5)	16 (61.5)	
N_2_	14		5 (35.7)	9 (64.3)		8 (57.1)	6 (42.9)		3 (21.4)	11 (78.6)	
**Tumor stage**											
I	14	**<0.0001**	3 (21.4)	11 (78.6)	0.798*	12 (85.7)	2 (14.3)	0.238*	6 (42.9)	8 (57.1)	0.413*
II	41		15 (36.6)	26 (63.4)		23 (56.1)	18 (43.9)		21 (51.2)	20 (48.8)	
III	29		9 (31.0)	20 (69.0)		20 (69.0)	9 (31.0)		10 (34.5)	19 (65.5)	
IV	11		3 (27.2)	8 (72.8)		7 (63.6)	4 (36.4)		3 (27.3)	8 (72.7)	
**Tumor grade**											
Low	75	**0.022**	22 (29.3)	53 (71.7)	0.362	47 (62.7)	28 (37.3)	0.303	27 (36.0)	48 (64.0)	**0.020**
High	20		8 (40.0)	12 (60.0)		15 (75.0)	5 (25.0)		13 (65.0)	7 (35.0)	

Median values were used as cut-off points for definition of subgroups (age group and tumor size).

*Fisher's Exact Test.

TSP50, testes-specific protease 50; CRC, colorectal carcinoma; CEA, carcinoembryonic antigen.

### Evaluation of TSP50 as potential diagnostic marker for CRC

**Figure 4:**
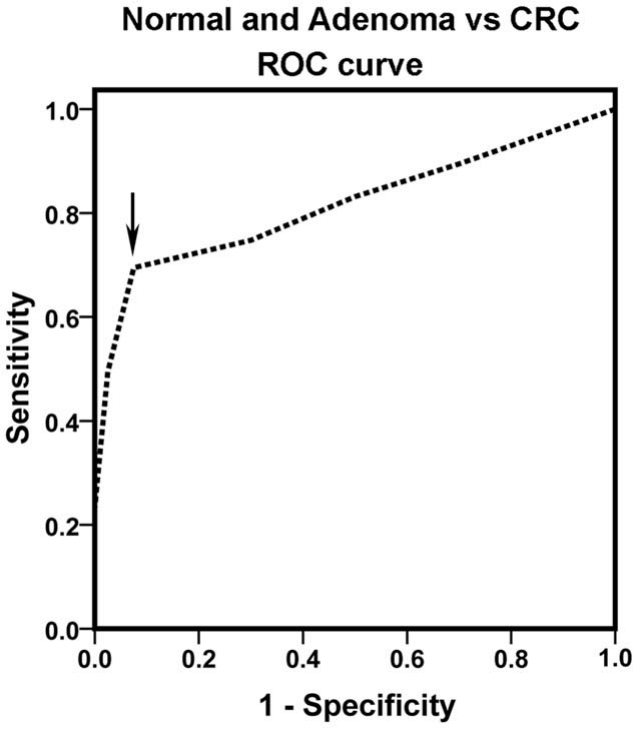
Receiver operating characteristic (ROC) curve of TSP50 in normal and adenoma vs CRC. Y-axis of the plot shows true-positive fraction (sensitivity) and X-axis shows false positive fraction (1-specificity). The arrow shows the part of the curve corresponding to the optimal cut-off values.

Receiver operating characteristic (ROC) analysis was used to determine the potential of TSP50 overexpression to distinguish CRCs from colorectal adenomas and normal tissues (Figure 4). The value of area-under-the-curve (AUC) was 0.812 (95% confidence interval (CI) = 0.741–0.883, *P*<0.001). Based on the best Youden index (the maximum value of [sensitivity + specificity – 1]) for TSP50, a cutoff score ≥4 (2+) was as positive criterion for statistical analysis of TSP50 immunostaining. The sensitivity, specificity, positive predictive value (PPV), negative predictive value (NPV) and Youden index were 68.4%, 92.5%, 95.6%, 55.2% and 60.9%, respectively (Table 3).

**Table 3:** Biomarker analysis of TSP50 in CRC.

Variables	Sensitivity	Specificity	PPV	NPV	Youden index	AUC (95% CI)
**Normal and adenoma vs.CRC**	68.4%	92.5%	95.6%	55.2%	62.0%	0.812 (0.741–0.883)

TSP50, testes-specific protease 50; CRC, colorectal carcinoma; PPV, positive predictive values; NPV, negative predictive values; Youden index was calculated as the maximum (sensitivity + specificity - 1); AUC, area under the curve; CI, confidence interval.

### Evaluation of TSP50 as potential prognostic marker for CRC

**Figure 5:**
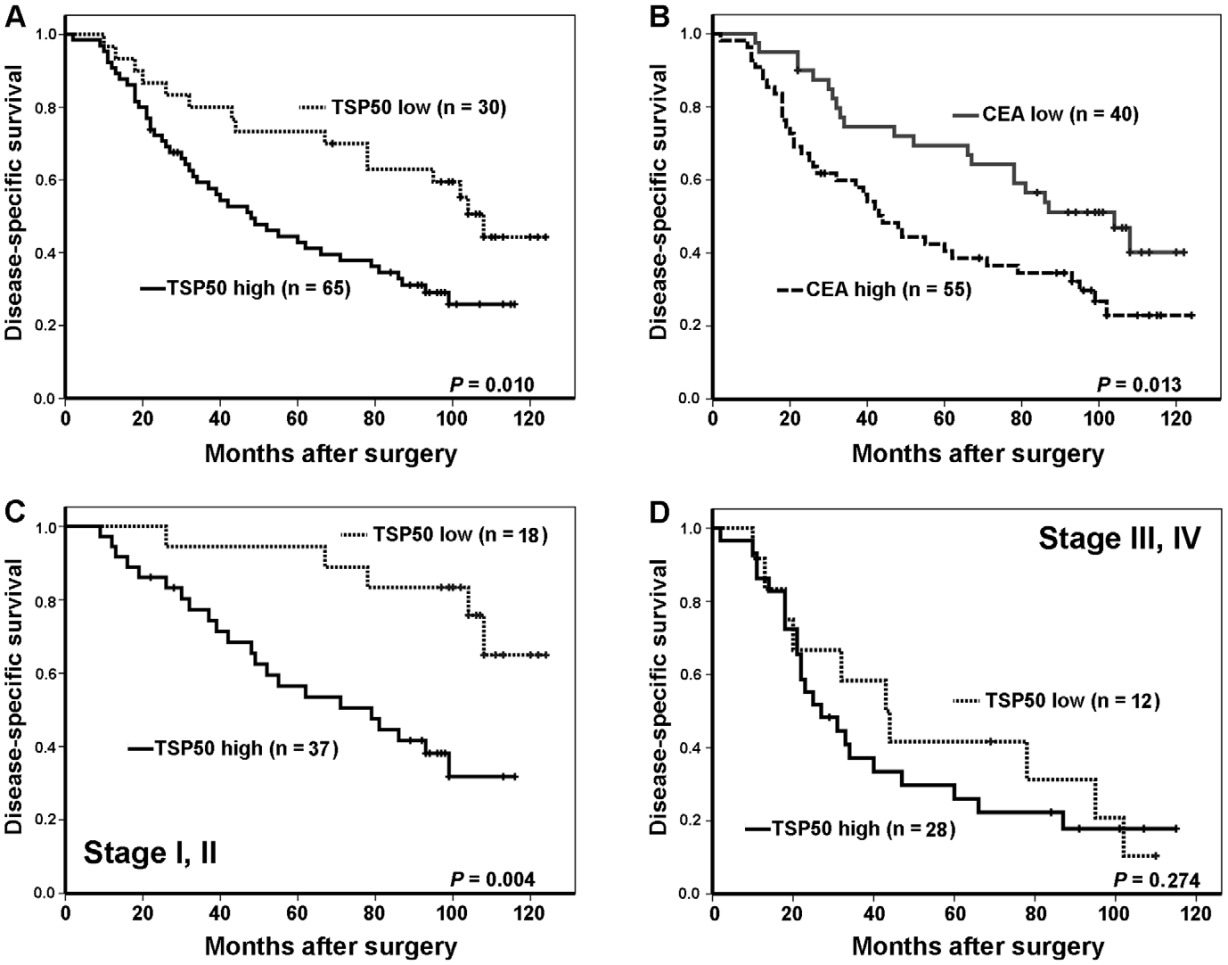
Kaplan-Meier survival curves illustrating the significance of TSP50 expression in comparison with CEA expression in CRC. (A) Overall, CRC patients with high TSP50 expression had shorter CRC-specific survival than those with low TSP50 expression (log-rank *P* = 0.010); (B) High CEA expression was associated significantly with poor CRC-specific survival relative to low CEA expression (log-rank *P* = 0.013); (C) In early-stage CRC (stage I and II), patients with high TSP50 expression had a significantly reduced CRC-specific survival relative to those with low expression (log-rank *P* = 0.004); (D) There was no significant difference between low and high expression of TSP50 in patients with advanced-stage CRC (stage III and IV; log-rank *P* = 0.274).

At the last follow-up, 60 of 95 patients (63.2%) had died from CRC, 29 of 95 patients (30.5%) remained alive, and 6 of 95 patients (6.3%) had died from other causes or lost touch. Univariate Kaplan-Meier survival analysis of the complete CRC patients (n = 95) based on TSP50 expression demonstrated that the disease-specific survival period was significantly shorter for patients with high TSP50 expression than for patients with low TSP50 expression (log-rank *P* = 0.010; Figure 5A). This result was similar with survival analysis based on CEA expression (log-rank *P* = 0.013; Figure 5B). Survival analyses, in early-stage (stage I and II) and advanced-stage (stage III and IV) group of CRC patients respectively, demonstrated that TSP50 overexpression was associated with shortened disease-specific survival for patients with early-stage CRC (log-rank *P* = 0.004; Figure 5C), but not for patients with advanced stage (log-rank *P* = 0.274; Figure 5D). Univariate Kaplan-Meier survival analysis based on clinicopathologic features showed that lymph node metastasis (log-rank *P*<0.0001), tumor stage (log-rank *P*<0.0001) and tumor grade (log-rank *P* = 0.022) were statistically significant risk factors affecting the disease-specific survival of CRC patients, except other clinicopathologic parameters (age, sex, tumor location, tumor size and depth of invasion; Table 2). In addition, high CEA expression was correlated with shorter survival for CRC patients (log-rank *P* = 0.013; Figure 5B), but a high or low expression of p53 was not related to the survival of CRC patients (data not show).

Cox regression analysis was carried out to evaluate the potential prognostic significance of TSP50 and CEA expression on CRC-specific survival in comparison with the clinicopathologic parameters. The backward stepwise multivariate regression analysis demonstrated that TSP50 expression, CEA expression and tumor stage were statistically significant independent prognostic indicators for CRC (Table 4).

**Table 4:** Backward stepwise multivariate regression analysis of prognostic factors.

Prognostic variables	Indicator of poor prognosis	HR (95% CI)	*P*-Value
**TSP50 expression: Low vs. high**	High expression	2.205 (1.214–4.004)	0.009
**CEA expression: Low vs. high**	High expression	1.813 (1.062–3.096)	0.029
**Tumor stage:**			
I vs II	II	1.988 (0.796–4.961)	0.141
I vs III	III	3.430 (1.380–8.526)	0.008
I vs IV	IV	18.781 (6.316–55.846)	<0.0001

Age, depth of invasion, lymph node metastasis, tumor size and tumor grade were excluded from the model because of *P*>0.05.

TSP50, testes-specific protease 50; CRC, colorectal carcinoma; CEA, carcinoembryonic antigen; HR, hazard ratio; CI, confidence interval.

## Discussion

This is the first study, to our knowledge, to report TSP50 expression in primary CRCs and evaluate its diagnostic and prognostic value. The salient findings of our study are: (a) TSP50 is abnormally highly expressed in CRCs in comparison with colorectal adenomas and normal tissues; (b) TSP50 expression is unrelated to p53 expression in CRC; (c) TSP50 overexpression distinguishes CRCs from colorectal adenomas and normal tissues with high specificity and PPV; and (d) high TSP50 expression in CRC is a novel independent factor for unfavorable prognosis.

Previous studies indicate that TSP50 is normally and specifically expressed in the spermatocytes of testes, and abnormally highly expressed in breast cancer cells and tissues, and it locates in the endoplasmic reticulum and the cytoplasm membrane [1], [2], [4]. In the present study, aberrant expression of TSP50 was found in the 7 CRC cell lines (Figure 1A and B), and its level was elevated in CRC compared with adjacent normal tissue (Figure1C). These results were confirmed by immunohistochemical analysis of CRCs, colorectal adenomas and normal tissues (Table 1). Similar to the earlier studies, TSP50 expression was observed predominantly in the cytoplasm of CRCs, and some CRC samples demonstrated membrane staining along with cytoplasmic localization (Figure 2J and K). Although an earlier investigation reported that TSP50 was not expressed in normal colon tissues by Northern blot analysis [1], weak expression of TSP50 in some normal colorectal tissues were observed by Western blot and immunochemical analysis in our study (Figure 1C and 2D). A possible explanation for this discrepancy is that previous investigation did not detect those normal colon tissues which weakly expressed TSP50. Interestingly, many other CTAs show low-level expression in a limited number of somatic tissues [16], [25].

We did not find any relationship between TSP50 expression and p53 expression by immunohistochemical analysis (Table 1). Antibodies used for immunohistochemistry can detect both the wild-type and the mutated p53 protein, and TSP50 expression is significantly higher in breast cancer cells in which *p53* gene is mutated [4], so TSP50 expression might be correlated with the status of *p53 gene* but not with the accumulated quantity of p53 protein in CRC. In this study, we found p53 expression was associated with tumor location as other authors did [26]. In contrast, some studies did not find any relationship to clinicopathological features [27]–[30], and others found a close relation to lymph node metastasis, invasion depth, distant metastasis or Dukes stage [31]–[34]. We did not find a correlation of p53 expression and prognosis, which is consistent with some previous studies [30], [35]–[39]. However, some investigations reported p53 expression had a better survival [26], [28], [40], and others reported poor prognosis in patients with p53-positive carcinomas [29], [34], [41]–[43]. Thus, the relationship between p53 expression and survival is still controversial. The discrepancies may result from different techniques used in these studies, such as different antibodies, scoring systems, cutoff-values and study populations. It has been found that *p53* gene encodes for at least ten different isoforms resulted from differential promoter utilization and alternative splicing [44], [45]. Each p53 isoform has different subcellular localisation and distinct biological activity, and some p53 isoforms were abnormally expressed in several tumor types [46]. Thus, it is proposed that some specific isoforms might be related to cancer prognosis. However, so far as we know, there is no report about the prognostic value of any specific isoform in CRC. Further investigations along this direction would open new perspectives for p53 studies. In addition, *p53* mutations, especially in exon 5 to 8 or codon 72, predicting poor survival in CRC patients are found by many studies [47]–[53], but it is still far from conclusion. Besides some contradictory results [54]–[56], the European Group of Tumor Markers (EGTM) and the American Society of Clinical Oncology (ASCO) did not recommend *p53* mutation detection for screening, diagnosis, staging, surveillance, determining prognosis or monitoring treatment of patients with CRC [57], [58]. On the whole, determination of the relationship between p53 status and cancer prognosis is much more complex than hitherto appreciated. It requires an integrated and complex analysis of p53 expression level, isoform type and gene mutation. 

The significant increase of TSP50 overexpression observed in CRCs (65 of 95 cases, 68.4%) as compared to colorectal adenomas and normal tissues (3 of 40 cases, 8%) is an important finding of our study, but there is no obvious correlation between TSP50 expression in CRCs and the clinicopathologic features (Table 2). In the further study of TSP50 diagnostic value for CRC, the ROC curve and Youden index were used for identifying the cutoff point at which optimal sensitivity and specificity were achieved, and the AUC showed the discriminatory power for TSP50 in CRCs. The high specificity and PPV but relatively low sensitivity and NPV indicate that TSP50 could accurately distinguish CRCs from colorectal adenomas and normal tissues but be not suitable for early screening of CRC. In addition, the value of Youden index and AUC demonstrate that this diagnostic method can be with relatively high validity and accuracy. TSP50 is hence an attractive and potential target for diagnosis and therapy.

In our study, survival analysis based on tumor stage (early and advanced stage) indicates that TSP50 is a prognostic factor of reduced survival in CRC patients, especially in those with early-stage tumors (stage I and II; Figure 5A and C). A statistically significant survival difference between high and low TSP50 expression was not observed for CRC patients in advanced stage (stage III and IV; Figure 5D), but studies with larger samples are needed to assess the prognostic importance of TSP50 expression in patients with CRC of this stage. Further, the multivariate Cox regression analysis demonstrated that increased expression of TSP50 is an independent indicator of unfavorable prognosis for patients with CRC (Table 4). Similarly, for a given cancer type, higher expression of some other CTAs is often correlated with worse outcome, such as MAGE-A3 for pancreatic cancer [59], MAGE-C2 for hepatocellular carcinoma [60], and NY-ESO-1 for malignant melanoma [61]. A recent study demonstrates that high TSP50 expression can promote tumorigenesis including cell proliferation, colony formation and migration [7], which may preliminarily explain the reason why TSP50 predict poor prognosis.

CEA is a widely accepted prognostic factor for CRC [57], [58], [62], [63]. Since the sample size was small, the CEA prognostic significance was tested in this cohort of CRC patients to verify their reliability. We examined CEA expression in tumor tissues (t-CEA) by immunohistochemical staining instead of preoperative CEA in serum (s-CEA), for the following two reasons: (a) for most patients in our study preoperative s-CEA was not detected; and (b) the prognosis value of t-CEA may be stronger than that of s-CEA in CRC due to the fact that level of s-CEA is affected by many factors, such as liver diseases, bowel obstruction and smoking, which could influence CEA production, release and metabolism [64]. Consistent with the earlier study, we found that t-CEA was also an independent predictor in this cohort of CRCs (Figure 5B; Table 4), and this result reveals that the cases selected are credible. In addition, it was found that well and moderately differentiated CRCs expressed increased t-CEA compared with poorly differentiated and undifferentiated tumors (*P* = 0.020). This finding is compatible with a report that s-CEA tends to be elevated in patients with well differentiated CRCs in comparison with poorly differentiated tumors [65].

In conclusion, we firstly report that TSP50 is abnormally and strongly expressed in CRCs, and it is a potential effective predictor for poor prognosis in CRC patients, especially for those at early stage. Though CRC is diagnosed on the basis of the results of colonoscopy or sigmoidoscopy with tumor biopsy [66], TSP50 might play a role on auxiliary diagnosis and become an attractive novel target for molecular imaging and therapy due to its high specificity and PPV for CRC. Determination of the TSP50 expression levels should help in identifying CRC patients with high risk, and that would be useful in the selection of patients for appropriate therapies. For example, the CRC patients with high TSP50 expression should accept a more aggressive treatment regimen and be followed-up carefully. Our findings remain to be validated in larger retrospective and prospective studies. More detailed elucidations of the function of TSP50 also require performing further molecular studies.

## Materials and Methods

### Ethic Statement

This study complied with the Helsinki Declaration and was approved by the Ethical Committee of Southwest Hospital of Third Military Medical University (Chongqing, China; Figure S1). Through the surgery informed consent form, our patients had already been informed that the resected specimens were kept by our hospital and might be used for scientific research but did not relate to patient's privacy. We further obtained the verbal consent of patients or their relatives by telephone during the follow-up.

### Cell lines and cell culture

CRC cell lines SW480, SW620, LoVo, HT-29, HCT 116, LS 174T and Caco-2 were obtained from the American Type Culture Collection. The breast carcinoma cell line MDA-MB-231 (a gift from Dr. Zhenning Tang, Breast Disease Center, Southwest Hospital, Third Military Medical University) was used as a postive control. The cells were cultured at 37°C in a humidified atmosphere of 95% air, 5% CO_2_ using DMEM (High Glucose) with 10% fetal bovine serum (Hyclone, Thermo Fisher Scientific, Waltham, MA, USA).

### RT-PCR

Total RNA was isolated from the cell lines by RNAiso Plus (TaKaRa Bio, Shiga, Japan). The first-strand cDNA was synthesized by ReverTra Ace -α- kit (TOYOBO, Osaka, Japan) following the manufacturer's instruction. The sense and antisense primers of TSP50 were 5′-CGCTCCTGTGGCTTTTCCTAC-3′ and GGAGGCGGTCTGCGTCAT-5′. The predicted size was 234 bp. β-actin was used as the internal control, the sense and antisense primers of which were 5′-ACCCCGTGCTGCTGACCGAG -3′ and 5′-TCCCGGCCAGCCAGGTCCA -3′. The predicted size was 249 bp. The PCR reaction mixture was comprised of cDNA derived from 200 ng of RNA, 2.5((l of 10(Ex Taq Buffer, 2((l of 25 mM MgCl2, 2((l of 10 mM deoxynucleotide triphosphates, 0.625 units of Ex Taq DNA polymerase (TaKaRa Bio), 10 pmol of sense and antisense primers from TSP50 or β-actin in a total volume of 25((l. PCR parameters were as follows: initial denaturation at 94(C for 5 min, followed by 30 cycles (denaturation at 94(C for 30 s, annealing at 55(C for 30 s, and extension at 72(C for 30 s) and final extension at 72(C for 5 min. The PCR products were separated on a 2% agarose gel. The experiments were done three times.

### Western blot analysis

Total protein in the cell lines and tissues (8 pairs of CRC and adjacent normal colorectal specimens from 8 patients randomly selected) was released by Ready Prep Protein Extraction Kit (Bio-Rad, Hercules, CA, USA). Protein concentration in each lysate was quantified using the bicinchoninic acid protein assay reagent kit (Pierce, Rockford, IL, USA). The total protein was subjected to 10% SDS/PAGE, and the resolved proteins were transferred electrophoretically to PVDF membranes (Millipore, Bedford, MA, USA). The membranes were blocked for 2 h with 5% non-fat milk in TBS buffer containing 0.05% Tween-20 (TBST) at 4°C, and then incubated with rabbit polyclonal antibodies to TSP50 (1∶500; Covalab, Cambridge, UK) and mouse monoclonal antibodies to β-actin (1∶400; Santa Cruz Biotechnology, CA, USA) respectively overnight at 4°C. After washing in TBST, the membranes were incubated with their respective secondary antibodies for 1 h, then incubated with SuperSignal West Femto Maximum Sensitivity Substrate (Pierce) for 1 min and imaged using a Gel Doc XR system (Bio-Rad). The experiments were done three times.

### Case selection and demographics

Ninety-five patients with primary CRC (mean age, 55 years old; age range, 23–82 years old) who underwent surgical resection at Southwest Hospital between 1997 and 2003 were identified. Patients who had a personal history of CRC or other malignancies were excluded. To control for treatment bias, the patients with CRC who were included were those who had undergone surgery and not received radiation therapy or presurgical chemotherapy across all tumor stages (stages I–IV). Postsurgical chemotherapies were performed depending on the severity of the disease and according to the National Comprehensive Cancer Network (NCCN) guidelines. Besides, 20 normal colorectal tissues from 20 body donors without intestinal disease, 20 colorectal adenomas from 20 patients and 3 breast cancer tissues from 3 patients before any anticancer therapy were collected. All the tissue blocks were formalin-fixed and paraffin-embedded (FFPE).

Patient demographics, along with clinical and follow-up information, were retrieved retrospectively from medical records and pathology reports. Through telephone and mail contacts, we ascertained outcome information directly from patients or relatives. Demographic data were collected, including patient age at diagnosis, sex, date of surgery, date of last follow-up (if alive) and date of death.

### Pathologic characteristics

Three pathologists (X.C.Y., G.J.D. and Q.L.W.) individually reviewed the surgical pathology reports and slides stained with hematoxylin and eosin for the degree of CRC histologic differentiation. The CRC tissues were regarded as well differentiated, moderately differentiated, poorly differentiated or undifferentiated according to the World Health Organization (WHO) guidelines. Reevaluation was necessary to reach a consensus when there was a disagreement among the three pathologists. The examined CRC cases were divided into two groups: the low-grade group, composed of well differentiated and moderately differentiated tumors, and the high-grade group, composed of poorly differentiated and undifferentiated tumors [67]. Pathologic staging was performed according to Union for International Cancer Control (UICC) criteria 7^th^ Edition. The anatomic locations of the CRC lesions were classified into two groups: the colon and the rectum. Three-dimensional tumor size was measured, and the largest tumor dimension was used for statistical analysis.

### Tissue microarrays and immunohistochemical staining

Ninety-five CRCs, 20 colorectal adenomas and 20 normal colorectal tissues were made into tissue microarrays using the tissue arrayer TMA-1 (Beecher Instruments, WA, USA) as described previously [68]. The breast cancer FFPE blocks were cut into 4-µm-thick sections. Immunohistochemistry was performed by a commercial streptavidin/peroxidase (SP) kit (Zymed, Invitrogen, Carlsbad, CA, USA) according to the manufacturer's instruction. In brief, the tissue microarrays and breast cancer sections were deparaffinized in xylene, hydrated in gradient alcohol, and pretreated in a microwave oven for 20 min in citrate buffer (0.01 M, PH 6.0) for antigen retrieval. The tissue microarrays and sections were incubated in 3% hydrogen peroxide at room temperature for 10 min to block endogenous peroxidase activity, and incubated with 10% goat serum at room temperature for 10 min to reduce nonspecific immunostaining. Then they were incubated with rabbit polyclonal antibodies to TSP50 (1∶400; Covalab), mouse monoclonal antibodies to p53 (1∶200; DO-7, Santa Cruz Biotechnology) or mouse monoclonal antibodies to CEA (1∶60; Col-1, Abcam, Cambridge, MA, USA). The primary antibody reaction was carried out at 4°C overnight. For a negative control, several breast carcinoma sections were incubated with PBS (0.01 mol/L, PH 7.4) instead of the primary antibodies. Sections were incubated for 30 min in respective secondary antibodies. Antigen–antibody complexes were colored by 3,3′-diaminobenzidine (Zymed, Invitrogen).

### Evaluation of immunohistochemical staining

Three pathologists (S.X., J.Z. and Q.W.) evaluated the immunostaining in a blinded fashion. If there was a discrepancy in individual evaluations, then all the three pathologists reevaluated the slides together to reach a consensus.

Immunohistochemical stainings of TSP50 and CEA were evaluated using a semi-quantitative scoring system for both staining intensity and the percentage of positive epithelial cells [69]. A score was calculated by multiplying the intensity (negative scored as 0, mild scored as 1, moderate scored as 2 and strong scored as 3) by percentage of stained cells (0, 0–10%; 1, 10–30%; 2, 30–50%; 3, 50–70%; and 4, 70–100%) [70], [71]. Scores of multiplication were graded as follows: −, 0; +, 1–3; ++, 4–8; +++, 9–12. p53 expression was evaluated according to the proportion of tumor cells with unequivocal strong nuclear staining, which was graded as follows: − (0–10%); + (11–49%); ++ (50%–74%); +++ (≥75%) [28], [37]. Additionally, for statistical analysis, the − and 1+ cases were pooled into the low-expression group, and the 2+ and 3+ cases were pooled into the high-expression group [72].

### Statistical analysis

The relationship between TSP50 expression and type of colorectal tissues or expression status of p53 or CEA was analyzed by Kruskal Wallis Test or Spearman's rho. Chi-square test was used to analyze the univariate associations of clinicopathological features with the expression status of TSP50, p53 or CEA. The statistical significance of each test was set at *P*<0.05. The ROC curve was used to calculate and quantify the sensitivity and specificity for CRC with respect to colorectal adenomas and normal colorectal tissues. The PPV, the NPV and Youden index (sensitivity + specificity - 1) were calculated.

The overall duration of survival was measured from the date of surgery to the date of death from CRC. Deaths were the outcomes (events) of interest. Those patients who died from causes other than CRC, lost contacts after last follow-up, or survived at the end of the study were considered to be censored. Survival curves were calculated using the Kaplan-Meier method in each group of patients with early-stage disease (stages I and II) and advanced-stage disease (stages III and IV), and differences were analyzed using the log-rank test. In addition to the primary analysis described above, Cox regression analysis was performed for backward stepwise multivariate analysis to find independent prognostic factors. The statistical significance of each test was controlled at *P*<0.05. All analyses were performed using the SPSS 17.0 (SPSS, Chicago, IL, USA).

## Supporting Information

Figure S1 **Statement of Ethical Committee.**
(TIF)
