# Supplementary figures and images for: High Expression of Testes-Specific Protease 50 Is Associated with Poor Prognosis in Colorectal Carcinoma
Source: PLoS One. 2011 Jul 12;6(7):e22203. doi: 10.1371/journal.pone.0022203 (PMC3134486; doi:10.1371/journal.pone.0022203)

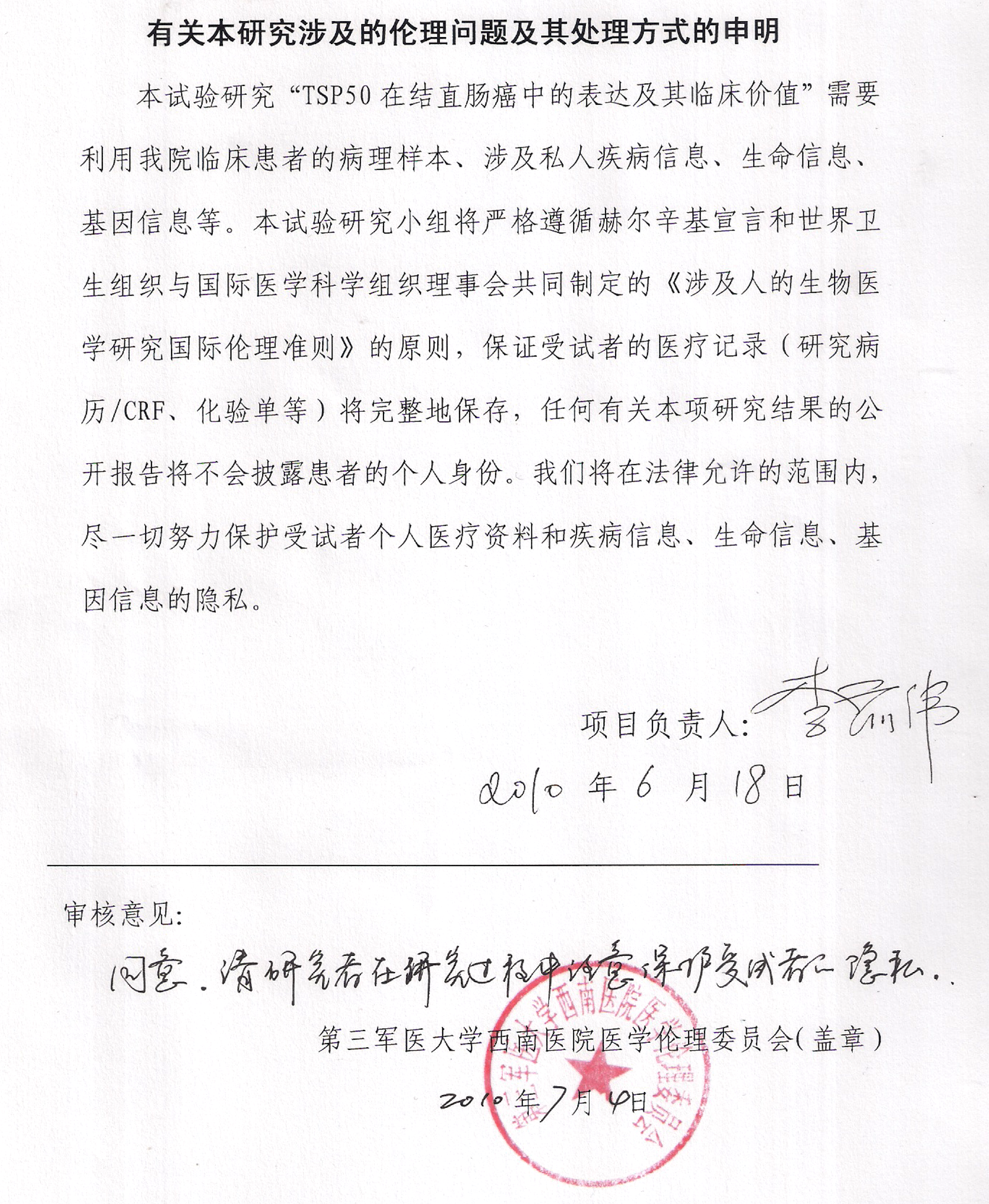

Supplement: Figure S1 — Statement of Ethical Committee. (TIF) [file pone.0022203.s001.tif]
